# Exploring the Rheological and Structural Characteristics of Novel Pectin-Salecan Gels

**DOI:** 10.3390/polym14214619

**Published:** 2022-10-31

**Authors:** Zhiping Fan, Ping Cheng, Lixia Chu, Jun Han

**Affiliations:** 1 Institute of BioPharmaceutical Research, Liaocheng University, Liaocheng 252059, China; 2 Liaocheng High-Tech Biotechnology Co., Ltd., Liaocheng 252059, China; 3 Business School, Liaocheng University, Liaocheng 252059, China

**Keywords:** hydrogels, rheology, salecan, pectin, polysaccharide

## Abstract

The hydrogels based on natural polysaccharide offers high hydrophilicity and excellent biocompatibility while exhibiting soft physical properties related to texture and tissues, making them ideal candidates for food and biomedical applications. Herein, a new gel system composed of pectin and salecan (PS) was designed and prepared, and its structural and functional characteristics were further explored by scanning electron microscopy and rheological testing. Data fitting based on Herschel–Bulkley (HB) and Power-Law models enable in-depth comparisons and elucidations of the PS gels’ flow behavior. The cyclic strain time scanning test gave an interesting maximum strain recovery rate of about 70%; meanwhile, the creep data reported an adjustable creep compliance of 0.0146 to 0.1802. The comprehensive analysis of the structure and rheological exploration of the novel pectin-salecan hydrogels demonstrated their potential advantages over pectin and broader applicability in different food or biomedical fields.

## 1. Introduction

Hydrogels obtained from natural polysaccharides, which are non-toxic, degradable, and easy to assemble in various combinations, could imbibe a substantial quantity of water/liquid in their networks without dissolving [1,2]. This feature ensures excellent biocompatibility and offers opportunities for being used in many important areas, including food and medicine [3,4]. Due to certain physical and chemical processes, the volume of the hydrogel or the gel-sol phase transition can change significantly. This characteristic will directly affect the release and flow behavior of nutrients or active ingredients, making the hydrogel a targeted delivery vehicle for nutrients/medicine and significantly influencing the rheology and texture of food.

Although biological characteristics such as biological toxicity and metabolism are certainly important for applying gel materials [5,6,7], rheological properties, such as flow behavior, viscoelasticity, and creep, have received more and more attention in polysaccharide-based hydrogels [8,9,10,11]. As the main method, at this stage, rheology provides researchers with a powerful tool to explore the flow behavior and structural properties of gels. Pectin is a biocompatible natural polysaccharide with a wide range of sources, usually extracted from plant fruits such as lemons, grapefruits, and apples [12,13]. It is an acidic polysaccharide whose backbone structure is made up of D-galacturonic acid connected by α-1,4 glycosidic bonds. Based on the extent of methoxylation, pectin is classified as low methoxyl (25–50%) and high methoxyl (50–80%) pectin [14]. Salecan is another novel water-soluble *β*-glucan (polysaccharide) fermented by the strain *Agrobacterium* sp. *ZX09*, which was discovered and industrialized in recent years with its newly registered CAS number 1439905-58-4. Although the traditional industry had proved that salecan is suitable for wastewater treatment (dye adsorption) [15], with the deepening of biomedical research, its new features, such as extraordinary solubility, excellent immunity improvement, antioxidant function, and outstanding rheological regulation performance, are gradually being recognized by researchers [16,17,18]. Considering the advantages of the two natural polysaccharides mentioned above, such as extensive sources and diverse functions, it is highly feasible to choose pectin and salecan to form a novel composite gel. This is of great significance for food and biomedical research, which can create new gel carriers for special cells/clinical nutrients, multifunctional hydrogel formulations, or even wearable gel biosensors for special areas.

The present work aims to prepare a novel pectin-salecan composite gel and conduct detailed structural and rheological studies combining various key rheological parameters and model-fitting data to facilitate its application in different fields.

## 2. Materials and Methods

Pectin was purchased from Anhui Yu Ning Pectin Co., LTD, China (Batch number: YNCP 401, 90% purity), and pure water was added to make 4% and 6% solutions, respectively; salecan (Synlight Bio, Batch number: C2101210901, 90% purity) was dissolved in water (S210, METTLER TOLEDO, Greifensee, Switzerland) at room temperature to form a 2% solution. In the low-concentration pectin (4%) group, when salecan: pectin is mixed at 1:1, 2:1, and 1:2, samples PSA, PSB, and PSC are obtained; in the high-concentration pectin (6%) group, when salecan: pectin is mixed at 1:1, 2:1, and 1:2, the samples are labeled as PSD, PSE, and PSF, respectively. The samples PSG and PSH are 4% and 6% pure pectin gel control groups, respectively. As a typical process, the two precursor solutions are mixed in a certain proportion, and calcium ions, which were obtained from Adamas-beta Chemical Co. (Shanghai, China), are added (final concentration 60 mM). After the mixture was incubated in a 90 °C water bath for 20 min, it was then placed at room temperature to stand overnight to obtain the final product. In order to carry out a series of rheological studies, such as creep testing, the linear viscoelastic region (LVR, 0.5% strain) of the samples was determined in advance as a reference. Detailed instructions on the preparation of PS system hydrogels and further characterizations can be found in “Supplementary Information”.

## 3. Results and Discussion

### 3.1. Hydrogel Design and Morphology Observation

**Figure 1:**
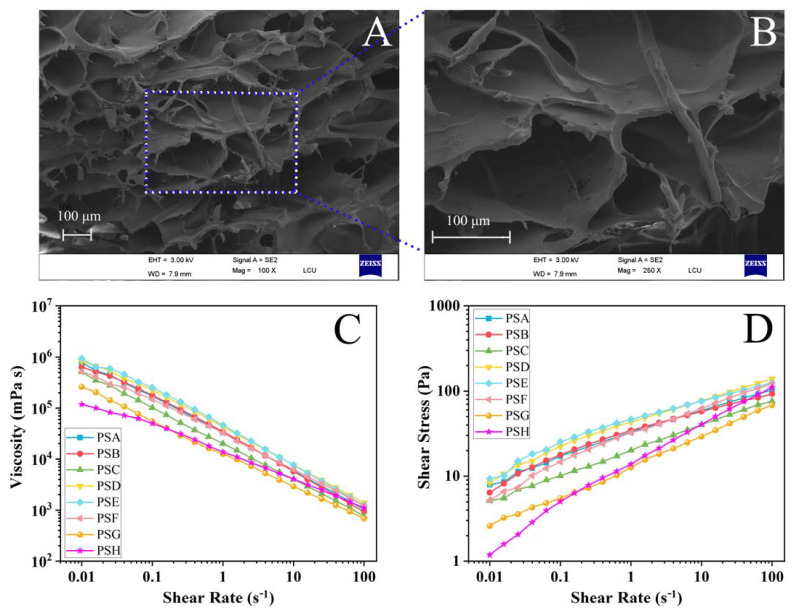
The SEM image of lyophilized PSA (The area within the dotted frame is the area for further magnification scanning) (**A**) and partial enlarged image (**B**); the viscosity (**C**) and shear Stress (**D**) curves in the pectin-salecan gels’ steady flow tests.

Final gels’ product quality is influenced by the ratio of composite hydrogels. A two-concentration pectin (4% and 6%) mixture with salecan (2%) was also tested in order to investigate the compounding effect under different concentrations. As a control group, two neat pectin hydrogels (PSG and PSH) of corresponding concentrations were also prepared. The interior microstructure of lyophilized PSA, which could potentially affect its biological properties, was examined using an electron microscope to determine its morphology. As shown in Figure 1A,B, the hydrogels are highly porous and well-connected, ranging in size from 0.05 to 80 microns based on random selection. This property is thought to be conducive to active ingredients encapsulation, as well as providing favorable conditions for nutrient transport [19,20]. Although the SEM morphologies of hydrogel samples may be similar, how to analyze the subtle differences between the samples’ morphologies and try to build the relationship between morphology and performance is also a challenging and worth-exploring topic.

### 3.2. Hydrogels Flow Behavior

With an increase in shear rate (Figure 1C,D), the composite hydrogels experienced an increase in shear stress as well as a decrease in viscosity. For exploring and analyzing a fluid’s or soft matter’s flow behavior, a variety of models can be used. Data were calculated and fitted using the Power-Law equation and the Herschel–Bulkley (HB) model, which helped in achieving the consistency coefficient (K), flow behavior index (n), and other parameters in this experiment.

There is evidence in the literature that the K value is influenced by viscosity and internal structure [21]. It is clear from the data fitting by the two models (Table 1) that the composite hydrogels’ values have a certain fluctuation range. The K value of a few samples (PSB and PSE) fluctuates, indicating that the applicability of the two models to the same sample is different, or it may be caused by the instability of the gels’ internal structure. It is widely regarded that substance flow characteristics can be evaluated using the flow behavior index (n). There is a common belief that pseudoplasticity increases as n reach zero in Newtonian fluids. From the listed data, no matter what model is used to fit the samples, the n values are significantly lower than 1. Notably, the two composite hydrogels, PSB and PSE with relatively low n values (0.149 and 0.158), are promising for food and pharmaceutical applications due to their ideal fluidity. A range of 0.996 to 0.999 is recorded for the correlation coefficients (R^2^) of pectin-salecan hydrogels obtained from both models. In this composite hydrogel system, although the fitting effect of both models is satisfactory, the HB model has a better degree of coincidence.

### 3.3. Creep Recovery Analysis

**Figure 2:**
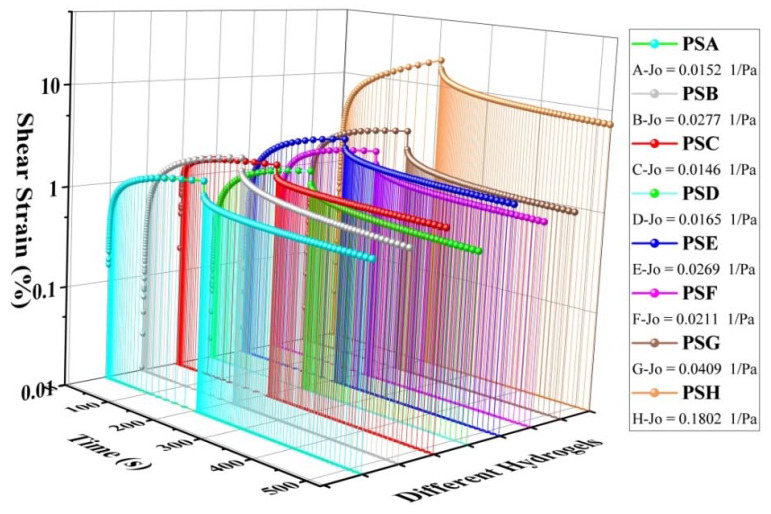
Pectin-salecan hydrogels’ creep recovery curves.

The evaluation of the pectin-salecan gels system should also consider creep recovery. A logarithmic increase in stress is applied to gel samples during this experiment; once it has been maintained for a period of time, it will be terminated immediately. Through the analysis of factors derived from the experiment, it is possible to quantitatively determine the recovery efficiency of understudy substances. It can be seen in Figure 2, the pure pectin gels PSG and PSH presented the most strain, whereas the composites pectin-salecan gels PSA and PSD were least affected. Their intricate crosslinking status, which is generally proved by Fourier Transform infrared spectrum data, is primarily responsible for this issue. As indicated by the test plots, the PSB and PSD samples show longer recovery periods after removing the applied stress than the PSC and PSE samples. The results indicate that PSB and PSD exhibit a partial viscous deformation, which resembles a viscoelastic fluid’s mechanical behavior. In contrast, the PSC and PSE underwent a small viscous deformation, resulting in shorter recovery times.

It is usually possible to define the equilibrium creep compliance (J_o_) as the ratio of G’ to G” corresponding to the terminal creep measurement area. According to the data, PSB and PSE exhibited the highest values of J_o_ among all the pectin-salecan gels, suggesting that these two gels are more capable than other samples of restoring viscoelasticity. Pectin-salecan interactions and binding efficiency are superior in certain specific situations, which may explain this fact.

### 3.4. Cyclic Strain Time Sweep

**Figure 3:**
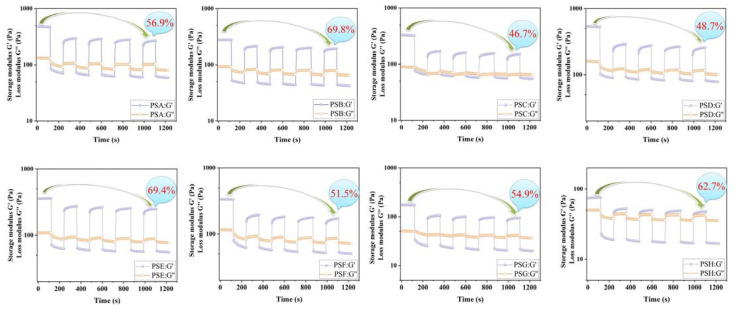
Pectin-salecan hydrogels’ cyclic strain time sweep.

Thixotropy reflects a special feature, that is, increasing the strain will greatly reduce the modulus of gels, and the modulus will quickly or gradually recover after the strain disappears. Cyclic strain time scanning is a general method to determine the thixotropy of samples by applying alternating large and small strains to gels. Figure 3 illustrated that, when PS hydrogels were exerted a small strain, they all displayed a typical elastic state (G’ > G”), whereas when they were exerted a large strain, PS gels showed a typical viscous state (G’ > G”)). After five cycles of strain application, the degree of modulus recovery of PS gels is significantly different due to different internal structures. As a consequence of differences in the pectin-salecan gels’ internal structures, modulus recovery after five cycles is significantly different. There are two top recovery rates of 69.8% and 69.4% for PSB and PSE gels, while the recovery rates of the remaining gels range from 46.7% to 62.7%. It is shown that, despite the ability of PS gels to recover partially from external forces, their integrity will still be partially compromised. Similar phenomena occur in other gel systems developed by our research group [22,23,24]. It can also be seen from Figure 3 that, compared with pure pectin gel, PSB and PSE gels have better recovery performance and are more suitable for advanced food formulations with high-flavor requirements.

## 4. Conclusions

A new gel system, consisting of pectin and salecan, was prepared by ionic crosslinking and heating methods. By using a scanning electron microscope, we were able to observe the structural characteristics of PS gels. While the Herschel–Bulkley and Power-Law models were then utilized to analyze the PS gels’ flow behavior. The cyclic strain time scanning test gave an interesting maximum strain recovery rate of about 70%; meanwhile, the creep data reported adjustable creep compliance of 0.0146 to 0.1802. All the comprehensive structural and rheological explorations demonstrated their potential advantages over pectin and broader applicability in some advanced food formulations or biomedical fields.

## Figures and Tables

**Table 1:** Model parameters fitted with the Power-Law and HB models.

Sample Label	Power-Law			HB Equation			
	K	n	R^2^	τ_o_	K	n	R^2^
PSA	34.297	0.239	0.999	6.063	38.742	0.226	0.999
PSB	36.110	0.205	0.999	9.754	54.018	0.158	0.999
PSC	21.919	0.273	0.996	1.55	20.259	0.295	0.999
PSD	42.621	0.257	0.999	3.527	57.085	0.207	0.999
PSE	46.681	0.215	0.999	9.769	76.368	0.149	0.999
PSF	32.174	0.293	0.998	6.256	37.996	0.263	0.999
PSG	12.549	0.371	0.997	0.705	11.8	0.385	0.999
PSH	15.079	0.435	0.999	0.989	15.469	0.429	0.999

## Data Availability

The corresponding author can provide any data in response to a request.

## Supplementary Materials

The following supporting information can be downloaded at: https://www.mdpi.com/article/10.3390/polym14214619/s1, Table S1: Composition of hydrogel samples.
