# Supplementary material for: Exploring the Rheological and Structural Characteristics of Novel Pectin-Salecan Gels
Source: Polymers (Basel). 2022 Oct 31;14(21):4619. doi: 10.3390/polym14214619 (PMC9659178; doi:10.3390/polym14214619)
Supplement: Supplementary file 1 [file polymers-14-04619-s001.zip › polymers-1985062-supplementary.pdf]

Supplementary Information

Exploring the Rheological and Structural Characteristics of Novel Pectin-Salecan Gels

Zhiping Fan[1], Ping Cheng[2], Lixia Chu[3], Jun Han*[1]*

[1]*Institute of BioPharmaceutical Research, Liaocheng University, Liaocheng 252059, China*

[2]*Liaocheng High-Tech Biotechnology Co., Ltd, Liaocheng 252059, China*

[3]*Business School, Liaocheng University, Liaocheng 252059, China*

Experimental Section:

Materials and methods

1 Materials

Commercially available pectin with model YNCP 401 was supplied by Anhui Yu Ning Pectin Co., Ltd. Salecan from *Agrobacterium sp. ZX09* was purchased from Synlight Bio Co., Ltd. (Sichuan, China). Sodium hydroxide and calcium chloride were obtained from Adamas-beta Chemical Co (Shanghai, China), which were used without any further purification. And many other ingredients were provided by Sinopharm Chemical Reagent Co., Ltd (Shanghai, China).

2 Methods

2.1 Hydrogel design and preparation

In order to fabricate the hydrogel samples, two components of polymer solution were first designed. The concentration of Salecan solution was set at 2%, and pectin was designed at 4% and 6%. The dispersion of 4%, 6% salecan was prepared by

stirring for 30 minutes with distilled water to form a homogeneous solution. The 2% Salecan solution was formed with distilled water under a stirring rate of 300 rpm for 30 min at room temperature. Different samples are mixed by different concentrations of polymer solution in different proportions and calcium ions (final concentration 60mM). Eight composite samples (PSA, PSB, PSC, PSD, PSE, PSF, PSG, and PSH), which contain two control group samples (PSG and PSH), were prepared in the hot water bath (90 ºC) to trigger the crosslinking of all the hydrogels. Then, the diverse mixed samples were poured into molds of uniform size and allowed to be fully crosslinked at room temperature. The composition of hydrogels was listed in Table S1.

Table S1 Composition of hydrogel samples

Samples label	Concentration of Salecan (%)	Concentration of Pectin (%)	Salecan: Pectin Ratio	Temperature
PSA	2	4	1:1	90 ºC
PSB	2	4	2:1	90 ºC
PSC	2	4	1:2	90 ºC
PSD	2	6	1:1	90 ºC
PSE	2	6	2:1	90 ºC
PSF	2	6	1:2	90 ºC
PSG	-	4	-	90 ºC
PSH	-	6	-	90 ºC

2.2 Observation of morphology

Morphologies of final hydrogels were characterized by scanning electron microscopy (GeminiSEM 300, Zeiss) using composite PSA as an example. The sample was frozen in a -20 ˚C freezer and lyophilized in a lyophilizer (Benchtop Pro 31, Virtis). The lyophilized sample was then positioned on SEM stubs with adhesive tape and sputter coated for 1 min with a thin gold layer. The morphology of the

sample was viewed and pictured at 5 kV accelerating voltage. The pore size was analyzed by randomly determining the pore diameters in three different visual fields.

2.3 Steady flow behavior

Intending to investigate the fluidity performance of different component hydrogels under stable shear conditions, we set the shear rate at 0.01-100 and exposed all the samples to this test condition for data acquisition. Based on the obtained data, the Power Law equation and Herschel-Bulkley (HB) model equations are used to fit the relevant parameters.

Power Law model: $\tau = K\gamma^{n}$

Herschel-Bulkley model: $\tau = \tau_0 + K\gamma^{n}$

In the above two equations, τ represent shear stress (Pa), τ_0 represents yield stress (Pa), K is consistency coefficient (Pa·s), γ is the shear rate (s^{-1}) and n is flow behavior index (dimensionless).

2.4 Thixotropy assessment

With a view to insight into the self-recovery ability of the novel Pectin-Salecan composite hydrogels, cyclic strain time sweeps were performed. For this purpose, at a frequency of 10 Hz, 5 cycles of time sweep which containing ten measuring sections, were performed alternately with a low strain of 0.1% and a high strain of 100%.

2.5 Creep recovery test

All the composites hydrogels were subjected to a gradually increasing stress, which was maintained for 180 seconds at the maximum value of 3 Pa. After that, the stress is removed immediately. The strain recovery gained from the internal structure reconstruction of the hydrogel itself was measured, while the equilibrium creep compliance (J_o) of each hydrogel sample was calculated.
